# Has the threshold for revision surgery for adverse reactions to metal debris changed in metal-on-metal hip arthroplasty patients? A cohort study of 239 patients using an adapted risk-stratification algorithm

**DOI:** 10.1080/17453674.2019.1659661

**Published:** 2019-09-09

**Authors:** Gulraj S Matharu, Fiona Berryman, David J Dunlop, Andrew Judge, David W Murray, Hemant G Pandit

**Affiliations:** a Nuffield Department of Orthopaedics, Rheumatology and Musculoskeletal Sciences University of Oxford, Nuffield Orthopaedic Centre, Oxford;;; b Musculoskeletal Research Unit, Translational Health Sciences, Bristol Medical School, University of Bristol;;; c The Royal Orthopaedic Hospital, Birmingham;;; d Leeds Institute of Rheumatic and Musculoskeletal Medicine, Chapel Allerton Hospital and University of Leeds, Leeds, UK

## Abstract

Background and purpose — A risk-stratification algorithm for metal-on-metal hip arthroplasty (MoMHA) patients was devised by US experts to help clinicians make management decisions. However, the proposed algorithm did not cover all potential patient or surgical abnormalities. Therefore we adapted the US risk-stratification algorithm in MoMHA patients revised for adverse reactions to metal debris (ARMD) to determine the variability in the revision threshold, and also whether high-risk patients had inferior outcomes following revision.

Patients and methods — We analysed 239 MoMHA revisions for ARMD between 2001 and 2016 from 2 centres with pre-revision blood metal ions and imaging. Patients were stratified (low risk, moderate risk, high risk) using pre-revision factors (implant, radiographic, blood metal ions, cross-sectional imaging) by adapting a published algorithm. The risk categories for each factor were assessed against revision year, revision centre, and post-revision outcomes (re-revision surgery, and any poor outcome).

Results — Compared with hips revised before 2012, hips revised from 2012 onwards included more high-risk implants (44% vs. 17% pre-2012), high-risk radiographic features (85% vs. 69% pre-2012), and low-risk metal ions (41% vs. 19% pre-2012). 1 centre more frequently revised patients with high-risk implants (48% vs. 14%) and low-risk blood metal ions (45% vs. 15%) compared with the other. All these comparisons were statistically significant (p < 0.05). With the limited sample size available, implant, radiographic, blood metal ion, and cross-sectional imaging risk groups did not statistically significantly affect the rates of re-revision surgery or frequency of poor outcomes post-revision.

Interpretation — When applying the adapted risk-stratification algorithm the threshold for ARMD revision changed over time, presumably due to increasing evidence, patient surveillance, and investigation since 2012. Lower blood metal ion thresholds were used from 2012 for ARMD revisions; however, there was evidence that centres attached different importance to metal ions when managing patients. High-risk patients did not have inferior outcomes following ARMD revision.

Metal-on-metal hip arthroplasty (MoMHA) in the form of stemmed total hip arthroplasty (THA) and hip resurfacing were used in large volumes, but due to high implant failure rates they have since been abandoned (Smith et al. 2012a, 2012b). Adverse reactions to metal debris (ARMD) represent the commonest revision indication (Matharu et al. 2016c, 2017b). Many worldwide regulatory authorities have proposed guidance on managing MoMHA patients (MHRA 2012, 2017, FDA 2013). However, these guidelines are complex, and in some cases are contradictory and not supported by evidence (Matharu et al. 2018c). Managing MoMHA patients therefore remains difficult and sometimes controversial, even in multidisciplinary teams (Berber et al. 2016).

In 2014, Kwon et al. published a risk-stratification algorithm for managing MoMHA patients. This consensus statement was devised by numerous expert US surgeons using their experience and the available evidence, as there was limited high-quality evidence to produce formal management guidelines. The proposed algorithm provided clinicians with information to risk stratify patients (low-, moderate-, and high-risk groups) according to their pre-revision factors, including implant, blood metal ions, and imaging (Kwon et al. 2014), given that often patients have pre-revision factors in different risk categories (Berber et al. 2016, Hussey et al. 2016). However, this algorithm was not comprehensive enough to account for all potential patient or surgical abnormalities.

To our knowledge 1 study has attempted to risk stratify patients using this algorithm, with the authors observing that the blood metal ion risk stratification was useful for distinguishing between revised and unrevised patients with recalled articular surface replacement (ASR) devices (Hussey et al. 2016). How this algorithm performs in non-ASR implants is unknown. Furthermore, it is not known how this risk-stratification algorithm relates to outcomes following ARMD revision, with limited data on any prognostic factors of outcome following ARMD revision available (Matharu et al. 2018a). Such information would help define the threshold for recommending revision, and when counselling patients regarding the outcomes associated with further procedures.

Given the risk-stratification algorithm proposed by Kwon et al. (2014) did not cover all potential patient or surgical abnormalities, we applied an adapted version of the algorithm to a large cohort of MoMHA patients who had all already undergone revision for ARMD at 2 centres. Using this adapted algorithm we assessed whether: (1) the revision threshold for ARMD changed over time, (2) the revision threshold differed between centres, and (3) whether patients at higher risk prior to revision had inferior outcomes following ARMD revision.

## Patients and methods

We performed a retrospective cohort study of prospectively collected data from 2 specialist UK arthroplasty centres (Nuffield Orthopaedic Centre, Oxford and the Royal Orthopaedic Hospital, Birmingham). The study included patients with large-diameter (36 mm or above) MoMHAs undergoing revision surgery for ARMD between January 2001 and March 2016. Cases were identified from prospectively maintained institutional databases described previously (Matharu et al. 2014, 2016b, 2016c, 2017a). This study was registered with each institution’s review board, with all patients reviewed according to institutional follow-up protocols.

There were 346 revisions performed for ARMD, confirmed intraoperatively and histopathologically, which were eligible for this study. Comprehensive details of this cohort including the definitions for ARMD, preoperative investigations, intraoperative findings at revision, follow-up after revision surgery, and the outcomes following revision have been described (Matharu et al. 2019). Briefly, both centres were tertiary units with 16 surgeons performing all cases. All patients underwent clinical examination and radiographic assessment (standardized anteroposterior pelvic radiographs +/– lateral hip radiograph), and most underwent blood cobalt and chromium ion sampling and cross-sectional imaging (ultrasound and/or metal artefact reduction sequence magnetic resonance imaging). The decision to perform revision surgery was made by the patient’s surgeon based on symptoms and/or investigative findings. After revision, patients were reviewed, usually annually, which included examination, radiographs, and completion of the Oxford Hip Score (OHS) questionnaire. Further investigations were performed in symptomatic patients (including those with new groin/thigh pain, clicking/clunking, limping, instability), with these tests including bloods (inflammatory markers and metal ions) and cross-sectional imaging. The threshold for performing investigations in symptomatic patients following ARMD revision was considered on a case-by-case basis at the discretion of each surgeon, and where appropriate by the multidisciplinary team. The present study includes only the ARMD revision patients with both pre-revision blood metal ions and cross-sectional imaging available.

We applied an adapted version of the algorithm proposed by Kwon et al. (2014) to stratify patients revised for ARMD into 3 risk groups (low, moderate, and high) based on their pre-revision factors, namely implant, radiographic, blood metal ions, and cross-sectional imaging. We used the same risk stratification for blood metal ions and cross-sectional imaging as proposed by Kwon et al. (2014). However, the risk stratification for implant and radiographic factors required adaptation from the original publication due to difficulties in applying the proposed algorithm. For implant factors, large-diameter modular THAs and recalled implants appeared in both the moderate-risk and high-risk stratification groups in the proposed algorithm. As the failure rates for large-diameter THAs are greater than those for hip resurfacing, and that recalled implants should be considered high risk, we assigned these 2 features to the high-risk category (Langton et al. 2011, Smith et al. 2012a, 2012b). For radiographic factors, the original algorithm did not fully define a suboptimally positioned acetabular component, as information on version was lacking. We considered acetabular components malpositioned if 1 or both parameters were outside the recommended optimal zone (inclination 35°–55° and anteversion 10°–30°) (Grammatopoulos et al. 2010). The adapted algorithm for implant, radiographic, blood metal ions, and cross-sectional imaging risk stratification used in the present study is summarised in Table 1.

**Table 1.** Adaptation of the risk stratification of metal-on-metal hip arthroplasty patients originally proposed by Kwon et al. (2014)

Pre-revision factor	Low risk	Moderate risk	High risk
Implant	– Non-recalled hip resurfacing in men under 50 years with OA	– All other non-recalled hip resurfacing implants	– Large-diameter (≥ 36 mm) modular THA
			– Any recalled implants
Radiographic	– Optimal acetabular component position **^a^**	– Optimal acetabular component position **^a^**	– Suboptimal acetabular component position **^a^**
	– No implant osteolysis or loosening	– No implant osteolysis or loosening	– Any implant osteolysis and/or loosening
Blood metal ions ^b^	– Both under 3 ppb	– Either or both between 3 and 10 ppb	– Either or both above 10 ppb
Cross-sectional imaging	– Within normal limits	– ARMD without muscle/bone involvement	– ARMD with muscle/bone involvement
		– Simple cystic lesions or small cystic lesions without thick walls	– Solid or mixed lesions
			– Cystic lesions with thick walls

ARMD = adverse reactions to metal debris; OA = osteoarthritis; ppb = parts per billion; THA = total hip arthroplasty.

**^a^** Optimal position defined in methods section.

**^b^** Chromium and cobalt

When assessing revision thresholds over time it was necessary to group patients by the year of ARMD revision. In 2012 the Medicines & Healthcare products Regulatory Agency (MHRA) issued a Medical Device Alert for all MoMHAs (MHRA 2012) in light of the high-profile reports of increased revision rates for both large-diameter modular THAs and hip resurfacing (Smith et al. 2012a, 2012b). Therefore patients were categorised as revised before 2012 or from January 2012 onwards.

The 2 outcomes of interest following ARMD revision were: (1) re-revision surgery, and (2) a poor outcome. Re-revision surgery was defined as removal, exchange, or addition of any implant. A poor outcome was defined as 1 or more of the following: intraoperative complication, postoperative complication, further surgery or procedure (including re-revision), mortality within 90 days of surgery, and poor OHS (less than 27 out of 48) (Murray et al. 2007).

### Statistics

The level set for statistical significance for all analyses was p < 0.05. For numerical data either the median and interquartile range (IQR), or the mean and standard deviation (SD) or range were used depending on data distribution. The effect of the implant, radiographic, blood metal ion, and cross-sectional imaging risk groups on (1) the year of revision, (2) the centre performing revision, and (3) post-revision outcomes (re-revision surgery and poor outcomes) were assessed using either the chi-square test or 2-sided Fisher’s exact test. The latter was used only when any cell had an expected frequency under 5.

Implant survival analysis was performed using the Kaplan–Meier method using re-revision surgery as the endpoint. Patients not undergoing re-revision were censored at latest follow-up or death. Cox regression models including adjustment for differences in age and sex were used to examine the effect of the radiographic, blood metal ion, and cross-sectional imaging risk groups on the rates of re-revision surgery. These models were presented as hazard ratios with 95% confidence intervals (CI). The proportional hazards assumption was assessed using scaled Schoenfeld residuals and satisfied for all regression analyses.

### Ethics, funding, and potential conflicts of interest

This study did not require ethical approval as all metal-on-metal hip arthroplasty patients were reviewed as part of each institution’s routine follow-up arrangements, which were adapted in response to published recommendations from the United Kingdom MHRA, and following revision all patients were reviewed as per the standard institutional protocols. Therefore no patients were specifically recalled for the study. The study was funded by Arthritis Research UK (grant reference number 21006), the Royal Orthopaedic Hospital Hip Research and Education Charitable Fund, and the Orthopaedics Trust. This paper presents independent research funded/supported by the National Institute for Health Research (NIHR) Leeds Biomedical Research Centre (BRC). The views expressed are those of the author(s) and not necessarily those of the NIHR or the Department of Health and Social Care.

GSM has received financial support for metal-on-metal hip research work from Arthritis Research UK, the Orthopaedics Trust, the Royal College of Surgeons of England, and the Royal Orthopaedic Hospital Hip Research and Education Charitable Fund. GSM has also received personal fees for undertaking medicolegal work on metal-on-metal hips for Leigh Day. FB and DJD receive institutional research funding from Smith & Nephew Orthopaedics UK. FB’s salary is paid by a grant from Smith & Nephew Orthopaedics UK. AJ has received consultancy fees from Freshfields Bruckhaus Deringer, and is a paid member of the data safety and monitoring board for Anthera Pharmaceuticals. DWM and HGP are paid consultants for Zimmer Biomet, and both receive institutional research funding from Zimmer Biomet. HGP is also a paid consultant for Kennedys Law, Bristol Myers Squibb, Depuy Synthes, Medacta Int, and Meril Life. He has received institutional research grants from UKIERI, Charnley Trust, Depuy Synthes, Glaxo Smith Kline, and NIHR.

## Results

There were 239 MoMHAs revised for ARMD with blood metal ions and cross-sectional imaging performed prior to revision surgery that were eligible for inclusion (Table 2).

**Table 2.** Demographics of metal-on-metal hip arthroplasty patients undergoing revision surgery. Values are frequency (percentage) unless otherwise specified

Pre-revision details	Cohort revised (n = 239)
Mean age at revision, years (SD)	60 (11)
Female sex (%)	168 (70)
Bilateral MoM hips any (%)	93 (39)
Bilateral MoM hips revised for ARMD (%)	35 (15)
Mean time to revision in years for ARMD (SD)	7 (3)
Primary and revision centre same	182 (76)
Primary implant type	
Hip resurfacing	150 (63)
Total hip arthroplasty	89 (37)
Primary implant design	
BHR	104 (44)
Other (THR or HR)	51 (21)
Conserve HR	29 (12)
Corail-Pinnacle THR	29 (12)
Synergy BHR THR	26 (11)
Primary implant head size	
<46	80 (42)
46 mm	60 (31)
>46	52 (27)
Symptoms	
Local symptoms	221 (93)
Systemic symptoms	2 (0.8)
Blood metal ions:	
Median cobalt, µg/l (IQR)	1.9 (0.7–8.0)
Median chromium, µg/l (IQR)	3.5 (1.6–8.3)
Radiographs	
Mean cup inclination in degrees (SD)	49 (11)
Mean cup version in degrees (SD)	19 (10)
Cup malposition	135 (57)
Stem/head malposition	3 (1)
Loose cup	9 (4)
Loose stem	11 (5)
Lysis cup	101 (42)
Lysis stem	42 (18)
Neck thinning	34 (14)
Impingement	1 (0.4)
Heterotopic ossification	20 (8)
Any cross-sectional imaging	
Any abnormality (% of those with imaging)	202 (85)
Pseudotumors (PT)	
PT numbers	163 (68)
PT consistency (% of all PT)	
Cystic	71 (44)
Mixed	83 (52)
Solid	7 (4)
PT location (% of all PT)	
Anterior ± lateral	64 (40)
Posterior ± lateral	48 (30)
Anterior + posterior ± lateral	29 (18)
Other	20 (12)
Median PT volume, cm^3^ (IQR)	45 (13–130)
Other image abnormalities	
Effusion	44 (18)
Muscle atrophy/damage	17 (7)
Tendon abnormality/damage	13 (5)
Bursal distension/thickening	24 (10)

BHR = Birmingham Hip Resurfacing; HR = hip resurfacing; IQR = interquartile range; PT = pseudotumor; SD = standard deviation; THA = total hip arthroplasty.

Note that micrograms per litre (µg/l) and parts per billion (ppb) are equivalent units of measure (see Table 1).

### Revision thresholds over time

Of the 239 hips, 181 (76%) were revised from 2012 onwards and 58 (24%) were revised before 2012. Hips revised from 2012 onwards were more likely to have high-risk implants (high-risk implants 44% from 2012 onwards vs. 17% pre-2012; p = 0.001 for chi-square test of year of surgery vs. implant risk category), high-risk radiographic features (85% vs. 69% pre-2012; p = 0.01), and low-risk blood metal ions (41% vs. 19% pre-2012; p = 0.001) (Table 3). A statistically significant difference could not be demonstrated in the cross-sectional imaging risk of hips revised from 2012 onwards compared with before 2012 (p = 0.4).

**Table 3.** Adapted risk-stratification group in relation to year of revision surgery. Values are frequency (percentage)

Risk-stratification group		Revised before 2012	Revised 2012 onwards	
	Overall	(n = 58; 24%)	(n = 181; 76%)	p-value
Implant				0.001
Low	11 (5)	3 (5)	8 (4)	
Moderate	138 (58)	45 (78)	93 (51)	
High	90 (38)	10 (17)	80 (44)	
Radiographic				0.01
Low/moderate	46 (19)	18 (31)	28 (16)	
High	193 (81)	40 (69)	153 (85)	
Blood metal ions				0.001
Low	86 (36)	11 (19)	75 (41)	
Moderate	92 (39)	23 (40)	69 (38)	
High	61 (25)	24 (41)	37 (20)	
Cross-sectional imaging				0.4
Low	43 (18)	7 (12)	36 (20)	
Moderate	106 (44)	26 (45)	80 (44)	
High	90 (38)	25 (43)	65 (36)	

### Revision thresholds at different centres

Of the 239 hips, 166 (70%) were revised at centre 1, and 73 (30%) were revised at centre 2. Hips revised at centre 1 were significantly more likely to have high-risk implants (48% vs. 14%; p < 0.001) and low-risk blood metal ions (45% vs. 15%; p < 0.001) compared with centre 2 (Table 4, see Supplementary data). The radiographic risk (p = 0.1) and cross-sectional imaging risk (p = 0.5) of hips revised for ARMD were not statistically significantly different between the 2 centres.

### Effect of risk stratification on outcomes after ARMD revision

Mean follow-up after revision was 5 years (1–16). During follow-up 22 hips (9%) needed re-revision surgery for any indication, and 92 hips (39%) had a poor outcome. The cumulative implant survival following ARMD revision at 5 years and 7 years was 89% (CI 83–93; 65 hips at risk) and 86% (CI 75–92; 23 hips at risk) respectively.

A difference could not be demonstrated between the various risk categories (implant, radiographic, blood metal ion, and cross-sectional imaging risk) and the frequency of re-revision surgery or the frequency of poor outcomes (Table 5, see Supplementary data). In addition, there was no statistically significant difference in the hazard ratios between the various risk categories (implant, radiographic, blood metal ion, and cross-sectional imaging risk) and the rates of re-revision surgery (Table 6, see Supplementary data).

## Discussion

We applied an adapted version of the current risk-stratification algorithm (Kwon el al. 2014) to a large cohort of MoMHA patients revised for ARMD at 2 tertiary European centres over a 15-year period. There was evidence that the threshold for performing revision surgery for ARMD has changed over time but also differed between centres. However, with the limited study sample available we found no evidence that patients considered high risk pre-revision subsequently experienced worse outcomes following ARMD revision surgery compared with moderate-risk and low-risk patients.

We observed that revisions performed from 2012 onwards were more likely to include high-risk implants, high-risk radiographic features, and low-risk blood metal ions. However, cross-sectional imaging risk was similar before and after 2012. Differences we observed between the revision thresholds used over time are likely to reflect increasing evidence, patient surveillance, and investigation in more recent years. The 2012 MHRA alert (MHRA 2012) and registry studies highlighting increased revision rates for MoMHAs (Smith et al. 2012a, 2012b) changed how these patients were managed worldwide, with evidence that revision rates have increased since regular surveillance was recommended (Matharu et al. 2016c, 2017b, 2018b). Around 2012 was also the time when it was widely recognised that large-diameter MoM THAs had universally high revision rates, as prior to this the problems reported were mainly in hip resurfacings (Pandit et al. 2008, Grammatopoulos et al. 2009). These large-diameter THAs are a high-risk group in the adapted algorithm, thus explaining why we observed more high-risk implants revised since 2012. As the evidence evolved, the importance of optimal acetabular orientation for MoMHA success was recognised (Grammatopoulos et al. 2010). This again explains why more high-risk radiographic features were seen from 2012, with most high-risk features in our series being due to suboptimal acetabular orientation rather than other adverse radiographic features. As the understanding of blood metal ions improved, some surgeons gradually started revising symptomatic MoMHA patients with lower blood metal ions on the premise that early revision would improve subsequent outcomes (Grammatopoulos et al. 2009, De Smet et al. 2011). We observed similar findings with lower blood metal ion thresholds used from 2012.

The revision threshold varied between centres, with 1 centre more commonly revising high-risk implants and low-risk blood metal ions compared with the other. Difference in implant risk reflects 1 centre performing high numbers of large-diameter MoM THA (high risk in the adapted algorithm) in addition to hip resurfacing (Matharu et al. 2016b, 2017b), whilst the other centre exclusively performed resurfacing using non-recalled designs (low risk and moderate risk) (Matharu et al. 2016c). Our observation that centres attached different importance to metal ions when managing patients ultimately requiring revision is supported by previous findings. 10 MoMHA clinical scenarios were used to examine how multidisciplinary teams from 6 experienced international centres managed problematic MoMHA patients, with agreement being inconsistent when patients had raised or rising blood metal ions (Berber et al. 2016). This is not surprising given many studies have proposed that a variety of different blood metal ion thresholds below the MHRA recommended 7 parts per billion (ppb) limit are better for managing MoMHA patients (Hart et al. 2011, Van Der Straeten et al. 2013). Furthermore, recent evidence established that the primary function of blood metal ions was for identifying patients at low risk of ARMD rather than for diagnosing ARMD (Matharu et al. 2016a, 2016b, 2017a). There is also great variability in the blood metal ion concentrations of MoMHAs revised for ARMD at different centres (De Smet et al. 2011, Liddle et al. 2013, Pritchett 2014), which supports our findings. 1 study reported pre-revision blood metal ions from as low as a median of 4 ppb (De Smet et al. 2011), whilst another centre reported ions ranging between 17 and 136 ppb in ARMD revisions (Pritchett 2014).

With the limited study sample available and the relatively low number of re-revisions, the implant, radiographic, blood metal ion, and cross-sectional imaging risk groups did not statistically significantly affect outcomes following ARMD revision in our study. It is intuitive that MoMHA patients with grossly raised blood metal ions and those with substantial osteolysis and/or tissue destruction on imaging should have inferior outcomes following revision compared with patients without such features, especially given the poor outcomes reported after the early ARMD revisions (Grammatopoulos et al. 2009, De Smet et al. 2011). Although some single-centre studies have identified predictors of poor outcomes following ARMD revision, such as solid ARMD lesions, these studies were small and underpowered (De Smet et al. 2011, Liddle et al. 2013, Matharu et al. 2014). Our larger study, which used an adapted risk-stratification algorithm proposed by expert surgeons, suggests that patients considered high risk pre-revision do not necessarily have inferior outcomes following ARMD revision compared with individuals with lower pre-revision risk. However, given the original algorithm required adaptation we question its clinical utility, and we also acknowledge that our modified algorithm was not perfect. Therefore we recommend further research to develop clinically useful algorithms for managing patients with problematic MoMHAs and also to inform thresholds for recommending revision surgery in MoMHA patients, if indeed such thresholds truly exist. In the interim it is recommended that surgeons continue to make decisions on an individual case basis and by using the best available evidence.

This study has limitations. Its retrospective nature may introduce potential bias, for example when assessing the cross-sectional imaging reports; however, undertaking a prospective study to answer the same questions would take many years. We acknowledge that focusing on patients with ions and imaging is a limitation; however, this was inevitable given the retrospective nature of the study and that the diagnosis and investigation of ARMD evolved over time (Grammatopoulos et al. 2009, De Smet et al. 2011). It was necessary to modify some of the initially proposed risk categories, such as implant factors, given the potential overlap between risk groups. This may be considered a limitation. However, it would otherwise have been impossible to apply the algorithm clinically given the original algorithm did not comprehensively cover all possibilities, and the original authors did recognise that their algorithm would evolve over time (Kwon et al. 2014). Similarly, it was not possible to assign each patient to 1 global risk category given they often had pre-revision factors in different risk groups. This limitation of the algorithm was recognised previously (Hussey et al. 2016), and we propose that more information was available by assessing each pre-revision category (implant, radiographic, blood metal ions, and cross-sectional imaging) separately. Patients were grouped by year of revision because there were not enough patients undergoing surgery each calendar year for meaningful analysis. Although this may obscure some detail regarding when changes occurred over time, such analysis would only be possible with registry data, which would lack most of the pre-revision data (ions and imaging). Some of the proportions/frequencies assessed varied between the different risk subgroups even though a statistically significant difference could not be demonstrated (Tables 3–5). It is acknowledged this may be a reflection that the sample size and/or number of events was too low to detect a difference. Finally, our findings apply only to MoMHA patients revised for ARMD, and not to asymptomatic patients with MoMHAs given we did not include such a comparator. It was never the intention to include a non-revised patient group given the study aims, and a previous study has already assessed the original algorithm in non-revised patients (Hussey et al. 2016). However, it is important to acknowledge that without applying the risk-stratification groups to patients who did not undergo revision surgery, it is not possible to conclusively demonstrate that the threshold for revision surgery changed over time.

In summary, when applying the adapted risk-stratification algorithm we found the threshold for revision surgery changed over time, which is likely to reflect the increasing evidence, patient surveillance, and investigation of MoMHA patients since 2012. Although lower blood metal ion thresholds have been used since 2012 for ARMD revisions, the centres studied attached different importance to metal ions when managing patients, which is consistent with previous findings. With the sample size available we found no evidence that patients considered high risk pre-revision experienced worse outcomes following ARMD revision surgery compared with moderate-risk and low-risk patients. Therefore further research is required to inform thresholds for recommending revision surgery in MoMHA patients.

### Supplementary data

Tables 4–6 are available as supplementary data in the online version of this article, http://dx.doi.org/10.1080/17453674.2019.1659661

## Supplementary Material

Supplemental Material
