# Supplementary material for: Has the threshold for revision surgery for adverse reactions to metal debris changed in metal-on-metal hip arthroplasty patients? A cohort study of 239 patients using an adapted risk-stratification algorithm
Source: Acta Orthop. 2019 Sep 9;90(6):530–6. doi: 10.1080/17453674.2019.1659661 (PMC6844394; doi:10.1080/17453674.2019.1659661)
Supplement: Supplemental Material [file IORT_A_1659661_SM4580.pdf]

Supplementary data

Table 4. Adapted risk-stratification group in relation to revision centre. Values are frequency (percentage)

Risk-stratification group	Overall	Centre 1 (n = 166; 70%)	Centre 2 (n = 73; 30%)	p-value
Implant				< 0.001
Low	11 (5)	5 (3)	6 (8)	
Moderate	138 (87)	81 (49)	57 (78)	
High	90 (38)	80 (48)	10 (14)	
Radiographic				0.1
Low/moderate	46 (19)	27 (16)	19 (26)	
High	193 (81)	139 (84)	54 (74)	
Blood metal ions				< 0.001
Low	86 (36)	75 (45)	11 (15)	
Moderate	92 (39)	65 (39)	27 (37)	
High	61 (25)	26 (16)	35 (48)	
Cross sectional imaging				0.5
Low risk	43 (18)	30 (18)	13 (18)	
Moderate risk	106 (44)	70 (42)	36 (49)	
High risk	90 (38)	66 (40)	24 (33)	

Table 5. Effect of adapted risk-stratification group on the frequency of re-revision surgery and poor outcomes. Values are frequency (percentage)

Risk-stratification group	Overall	Re-revision (n = 22; 9%)	Poor outcome (n = 92; 39%)
Implant			
Low	11 (5)	2 (18)	5 (46)
Moderate	138 (87)	11 (8)	46 (33)
High	90 (38)	9 (10)	41 (46)
p-value		0.4	0.2
Radiographic			
Low/moderate	46 (19)	7 (15)	18 (39)
High risk	193 (81)	15 (8)	74 (38)
p-value		0.2	0.9
Blood metal ions			
Low	86 (36)	6 (7)	30 (35)
Moderate	92 (39)	6 (7)	37 (40)
High	61 (25)	10 (16)	25 (41)
p-value		0.1	0.7
Cross-sectional imaging			
Low	43 (18)	5 (12)	17 (40)
Moderate	106 (44)	6 (6)	33 (31)
High	90 (38)	11 (12)	42 (47)
p-value		0.2	0.1

Table 6. Effect of adapted risk-stratification group on the rates of re-revision surgery

Risk-stratification group	Cox regression hazard ratio (95% confidence intervals)
Implant	
Low	Reference group
Moderate	0.4 (0.1–1.7)
High	0.6 (0.1–2.7)
Radiographic	
Low/moderate	Reference group
High risk	0.6 (0.2–1.4)
Blood metal ions	
Low	Reference group
Moderate	0.8 (0.3–2.5)
High	2.1 (0.7–5.8)
Cross-sectional imaging	
Low	Reference group
Moderate	0.4 (0.1–1.3)
High	1.0 (0.3–2.9)
